# Prognostic Comparison of Complete vs. Incomplete Radiofrequency Ablation for Colorectal Liver Metastases: A Multicenter Prospective Study

**DOI:** 10.1002/cam4.70735

**Published:** 2025-04-15

**Authors:** Huilin Lu, Xuancheng Xie, Yulan Zeng, Xiangwen Xia, Xiangjun Dong, Futang Bu, Hongjie Fan, Shufeng Xu

**Affiliations:** ^1^ Department of Interventional Therapy Xinxiang Central Hospital/the Fourth Clinical College of Xinxiang Medical University Xinxiang Henan China; ^2^ Department of Radiology The First People's Hospital of Yunnan Province Kunming Yunnan China; ^3^ Cancer Center, Union Hospital, Tongji Medical College Huazhong University of Science and Technology Wuhan China; ^4^ Department of Radiology, Union Hospital, Tongji Medical College Huazhong University of Science and Technology Wuhan Hubei China; ^5^ Hubei Provincial Clinical Research Center for Precision Radiology & Interventional Medicine Wuhan China; ^6^ Hubei Provincial Key Laboratory of Molecular Imaging Wuhan China; ^7^ Department of Radiation Oncology, Zhongshan Hospital Fudan University Shanghai China; ^8^ Department of Radiology, The Quzhou Affiliated Hospital of Wenzhou Medical University Quzhou People's Hospital Quzhou China

**Keywords:** colorectal liver metastases, computed tomography, local tumor progression‐free survival, new intrahepatic metastases, overall survival, radiofrequency ablation

## Abstract

**Background:**

Radiofrequency ablation (RFA) is a curative treatment for colorectal liver metastases (CLMs) in selected patients. NCCN guidelines recommend RFA for both unresectable and select resectable CLMs when complete ablation with adequate margins is feasible. While RFA can achieve oncologic outcomes comparable to surgery in well‐selected patients, residual tumors are associated with a poorer prognosis.

**Objectives:**

To identify predictors of residual tumor after percutaneous RFA for CLMs and evaluate their impact on overall survival (OS) and new intrahepatic metastases (NIHM).

**Methods:**

We prospectively included patients with CLMs who underwent percutaneous RFA from November 2019 to November 2022. Dynamic contrast‐enhanced computed tomography assessed CLMs before and after RFA. Residual tumor was defined as active tumor visible immediately post‐ablation or within 4–8 weeks, within 1 cm of the ablation zone. Data from three centers formed a developmental cohort, validated with patients from a fourth center. Cox regression and Kaplan–Meier analysis assessed local tumor progression‐free survival (LTPFS), NIHM, and OS.

**Results:**

Among 200 patients (mean age 61 years, 126 men) with 410 tumors, independent predictors of residual tumors included perivascular tumor location (odds ratio [OR] = 6.673), tumor size ≥ 20 mm (OR = 3.925), and minimal ablative margin (OR = 0.599). These factors also predicted LTPFS. NIHM was more frequent in the residual tumor group than in the complete RFA (cRFA) group (*p* = 0.002). Median OS was 45 months, shorter in the residual tumor group (30 vs. 48 months, *p* = 0.009). Patients with NIHM who received transarterial chemoembolization combined with hepatic arterial infusion chemotherapy had a median OS of 43 months, compared to 34 months with RFA alone (*p* = 0.039).

**Conclusions:**

A non‐perivascular tumor location, tumor size < 20 mm, and a sufficient ablation margin are essential for achieving complete RFA. Residual tumors are associated with increased NIHM and shorter OS.

**Abbreviations**
AUC: area under the curve
CLM: cancer liver metastases
CRC: colorectal cancer
CT: computed tomography
DCE: dynamic contrast‐enhanced
GLM: generalized linear model
HAIC: hepatic arterial infusion chemotherapy
LTPFS: local tumor progression‐free survival
MLR: multivariate logistic regression
MR: magnetic resonance
MWA: microwave ablation
NIHM: new intrahepatic metastases
OR: odds ratio
OS: overall survival
RFA: radiofrequency ablation
ROC: receiver operating characteristic
TACE: transarterial chemoembolization

## 1 Background

Colorectal cancer (CRC) is a major contributor to the global cancer burden, ranking third in morbidity and second in mortality among all cancers [1, 2]. The liver is the most common organ for metastases, with over 25% of patients with CRC developing liver metastases during their disease course [3, 4, 5]. Although surgery is considered the optimal intervention for patients with colorectal cancer liver metastases (CLMs), fewer than 20% of these patients are eligible for surgical resection [6]. Minimally invasive alternatives, such as selective ablation, are increasingly used for both resectable and unresectable tumors, particularly when small tumors can be treated with adequate margins [7, 8, 9]. Ablation can be used alone or in combination with resection if all visible disease is eradicated, offering satisfactory quality of life and long‐term overall survival (OS) for affected patients [7, 9]. Research suggests that RFA for CLMs achieves 15‐year local tumor progression‐free survival (LTPFS) and OS rates of 72% and 28%, respectively, highlighting its long‐term effectiveness in controlling CLMs and establishing it as a viable alternative to surgery for small CLMs [8]. Therefore, in the treatment of CLMs, experts emphasize the critical role of interventional oncology, particularly ablation techniques [9]. This emphasis has led to a shift in terminology from “resectable disease” to the more inclusive term “locally treatable disease” [9].

In vivo, microwave ablation (MWA) does not rely on tissue conductivity and is less affected by tissue carbonization, dehydration, and the cooling effect caused by blood flow perfusion [10, 11]. In contrast, the heat sink effect presents a significant limitation for RFA, leading to higher local tumor recurrence rates in perivascular tumors. Although percutaneous balloon occlusion can mitigate this effect and reduce LTP rates to levels comparable to those of non‐perivascular tumors, its widespread adoption in clinical practice remains challenging [10, 11]. The effectiveness of RFA in treating CLMs is influenced by various tissue characteristics, including thermal conductivity, peripheral blood vessels, and water content [12]. When a tumor is located near the main blood vessels of the porta hepatis, the heat sink effect disrupts temperature distribution, reducing the therapeutic efficacy of RFA. Additionally, factors such as reduced energy delivery to avoid thermal damage, unpredictable ablation range, and electrode positioning errors can contribute to incomplete RFA, resulting in residual tumors. These residual tumors may lead to early new intrahepatic metastases (NIHM) and poor OS in patients with CLMs [13]. This process may involve the activation of proteins (e.g., phosphoproteins and heat shock proteins [14, 15]) and signaling pathways (e.g., the STAT3/c‐Met pathway [16]), which are associated with biological mechanisms such as inflammation, autophagy, and hypoxia [13, 17].

Previous studies have analyzed the oncological outcomes of RFA in treating CLMs, indicating that tumor size and minimal ablative margin are independent predictors of both LTPFS and OS [6, 8, 18]. Additionally, some studies have shown that the subcapsular location of tumors and their proximity to blood vessels serve as independent risk factors for LTPFS [8, 10]. However, to date, no predictive model for residual tumor of CLMs has been reported, nor has a multicenter, prospective cohort study on this topic been conducted. Therefore, our aim was to develop and validate a predictive model for residual tumor and evaluate its effect on NIHM and OS.

## 2 Materials and Methods

### 2.1 Study Population

This study was approved by the Ethics Review Committee of our hospital, and the study population consisted of patients with CLMs who underwent percutaneous RFA at four medical institutions between November 2019 and November 2022. The inclusion criteria were as follows: (1) no uncorrectable coagulation disorders; (2) maximum diameter of CLMs not exceeding 50 mm, with no more than five lesions; (3) the primary tumor had been surgically removed; (4) liver metastases had not previously received interventional treatments (such as transarterial chemoembolization [TACE], hepatic arterial infusion chemotherapy [HAIC], or any form of ablation); (5) no significant organ failure, including heart, kidney, or liver failure; and (6) no biliary infections or sepsis.

During follow‐up and data collection, the exclusion criteria were as follows: (1) pathological diagnosis not confirming CLM; (2) palliative or staged RFA treatments; and (3) absence of essential clinical or imaging data during the preoperative, postoperative, or follow‐up periods.

### 2.2 RFA Procedure

A whole‐liver dynamic contrast‐enhanced (DCE) scan was obtained using a 64‐row spiral computed tomography (CT) scanner (GE LightSpeed VCT, Boston, Massachusetts, USA) 3 days before RFA. The scanning and reconstruction parameters are provided in the Table S1. Routine preoperative laboratory tests were performed to assess for contraindications. Percutaneous intrahepatic RFA was performed using the RF‐3000 radiofrequency ablation system (Boston Scientific Corporation, Boston, USA). For percutaneous infiltration anesthesia, 5–10 mL of 2% lidocaine (North China Pharmaceutical Co. Ltd., Hebei, China) was administered, and hydromorphone hydrochloride (Renfu Pharmaceutical Co. Ltd., Hubei, China) was provided as an analgesic. A 20‐row spiral CT scanner (Siemens Definition AS, Erlangen, Germany) was used to guide the radiofrequency electrode to the edge of the lesion and to deploy the sub‐electrodes. RFA was initiated once the sub‐electrodes adequately covered the lesion, ensuring that the ablation zone extended at least 5 mm beyond the tumor margin into the surrounding liver parenchyma. Upon electrode withdrawal, the needle tract was cauterized to prevent tumor seeding or bleeding. A post‐RFA DCE‐CT scan was performed immediately to evaluate ablation coverage and detect potential complications.

### 2.3 Definition

Residual tumor was defined as a viable tumor visible either immediately after ablation or on the first imaging examination (within 4–8 weeks post‐ablation), located within 1 cm of the original ablation zone. If no residual tumor was detected, the procedure was classified as complete radiofrequency ablation (cRFA). CLMs adjacent to the first‐ or second‐order branches of the portal or hepatic vein, with a vessel diameter of ≥ 3 mm, were classified as perivascular tumors. Subcapsular CLMs were defined as tumors situated less than 5 mm from the hepatic capsule. Following a previously reported method [6, 8, 18, 19], the distance from the tumor edge to a selected anatomical landmark was measured on pre‐RFA DCE‐CT images in the transverse, sagittal, and coronal planes. The corresponding distance from the ablation edge to the same landmark was measured using the same three tomographic DCE CT images 1 month after RFA. Ablative margin values were calculated by subtracting the post‐RFA ablation boundary‐to‐landmark distance from the pre‐RFA tumor boundary‐to‐landmark distance. The smallest of these values was defined as the minimal ablative margin achieved by RFA. For subcapsular CLMs, the ablation defect in the liver parenchyma at the tumor margin was measured during the RFA procedure. To assess the minimal ablative margin for subcapsular CLMs, the study measured the liver parenchyma defect caused by RFA adjacent to these tumors. NIHM was defined as new intrahepatic metastases distinct from local tumor recurrence, which were characterized as recurrence occurring within or up to 1 cm around the ablation zone.

### 2.4 Follow‐Up

All patients underwent follow‐up evaluations within 4–8 weeks after RFA to assess treatment efficacy. Repeat RFA was performed for incompletely ablated tumors identified on DCE‐CT. In cases of intrahepatic tumor progression, repeat interventional therapies (such as SBRT, TACE, HAIC, or RFA) were considered as needed. OS was calculated from the date of RFA to the date of patient death, while the interval of NIHM was defined as the time from RFA to its identification. LTPFS was defined as the time from RFA to radiological evidence of LTP or the last follow‐up. The primary endpoints of the study were residual tumor, LTPFS, NIHM, and OS. The secondary endpoint was the choice of interventional treatment modalities for residual tumor, LTP, or NIHM.

### 2.5 Statistical Analysis

We (H.L. and X.X.) conducted statistical analyses using R 4.0.2 [20], Stata 17.0 (StataCorp., TX, USA), and SPSS 24.0 (IBM, Chicago, USA). Continuous variables (e.g., age) following a normal distribution were compared using Student's *t*‐test, while categorical variables (e.g., sex) were analyzed using the *χ* ^2^ test. Based on previous studies, we assumed an incidence of residual tumors of 10%. We planned to include four independent variables in the multivariate logistic regression model, with at least 8–10 residual tumor cases per variable, while considering a 10% loss to follow‐up. Based on these assumptions, we estimated that approximately 200 patients or 400 CLM samples would need to be included.

This study aimed to develop and validate a robust predictive model for residual tumor, LTPFS, and OS, which involved three main steps. Initially, independent variables were selected based on literature review, expert input, clinical relevance, and statistical significance among groups. Subsequently, predictive models were developed using data from three initial centers and validated with patients from a fourth center. In the development dataset, potential risk factors were identified through univariate analysis, and a multivariate logistic regression (MLR) model was adjusted to minimize overfitting, considering the residual tumor group sample size and univariate analysis results. An omnibus test compared the model's probability distribution with that of a null model, while the Hosmer–Lemeshow test was used to assess model fit. Receiver operating characteristic (ROC) curves, including area under the curve (AUC) calculations, were used to evaluate model performance.

Additionally, univariate and multivariate Cox proportional hazards regression analyses were conducted to identify factors influencing LTPFS. Patients who died before experiencing LTP or NIHM were treated as competing risk events. After adjusting for competing mortality, a multivariate Cox regression analysis was performed to identify independent predictors of OS. Furthermore, based on follow‐up data on LTP and NIHM, differences in OS among patients with CLM treated with various therapies were analyzed using pairwise Kaplan–Meier survival analysis and log‐rank tests. These methods were also used to estimate and compare NIHM and OS outcomes between the residual tumor and cRFA groups. Statistical significance was defined as *p* < 0.05.

## 3 Results

### 3.1 Patient Characteristics

**FIGURE 1:**
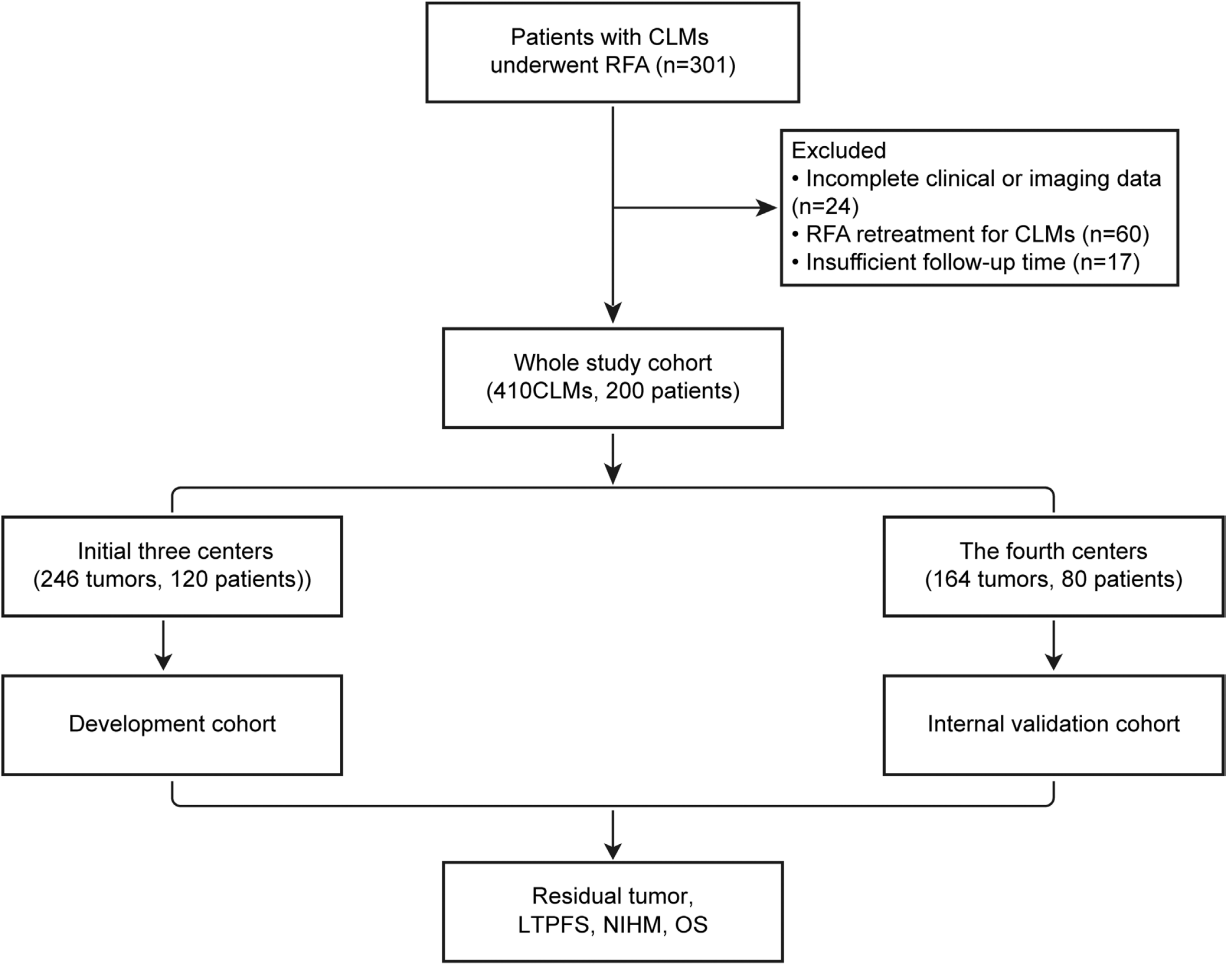
Flow diagram of the study design. CLMs, colorectal cancer liver metastases; LTPFS, local tumor progression‐free survival; NIHM, new intrahepatic metastases; OS, overall survival; RFA, radiofrequency ablation.

During the study period, a total of 301 patients with CLMs underwent RFA, of whom 101 were excluded due to insufficient data (*n* = 41) or repeated RFA (*n* = 60) (Figure 1). The final study cohort included 200 hospitalized patients with 410 CLMs. The mean age of these patients was 61.0 ± 10.5 years (range, 27–89 years), with males comprising 63.0% (Table 1). The most common primary tumor location was the sigmoid colon (65/200), followed by the rectum (57/200). Approximately half of the patients had synchronous CLMs (106/200), and 72 patients (36.0%) had extrahepatic metastases, most frequently in the lungs. Comorbidities were observed in 101 patients (50.5%), with hypertension being the most common. Pre‐RFA laboratory tests showed elevated carcinoembryonic antigen (> 30 μg/mL) in 45 patients (22.5%) and elevated carbohydrate antigen 19‐9 (> 37 U/mL) in 55 patients (27.5%). Fifty‐five patients (27.5%) had previously undergone liver resection, and 172 patients (86.0%) had received chemotherapy before RFA. Major RFA‐related complications occurred in 76 cases (38.0%), primarily pain (54 cases, 27%), bleeding (19 cases, 9.5%), and pleural effusion (3 cases, 1.5%). No significant differences in baseline characteristics were found between the two cohorts (Table 1).

**TABLE 1:** Clinicopathological and demographic characteristics of patients in the development and internal validation cohorts.

	Development set	Validation set	t/*χ* ^2^	*p*
Patients	120	80	—	—
Tumors	246	164	—	—
Age (years)	60.6 ± 11.0	61.7 ± 9.6	−0.715	0.475
Sex			1.033	0.309
Male	79 (65.8)	47 (58.8)		
Female	41 (34.2)	33 (41.3)		
BMI (kg/m^2^)	22.9 ± 2.5	23.5 ± 3.2	−1.588	0.154
Primary tumor location			5.687	0.224
Rectum	30 (25.0)	27 (33.8)		
Sigmoid colon	42 (35.0)	23 (28.8)		
Descending colon	13 (10.8)	14 (17.5)		
Transverse colon	9 (7.5)	6 (7.5)		
Ascending colon	26 (21.7)	10 (12.5)		
Differentiation (missing, *n* = 25)			2.924	0.571
Low	15 (15.2)	10 (13.2)		
Low to medium	8 (8.1)	8 (10.5)		
Medium	34 (34.3)	32 (42.1)		
Medium to high	29 (29.3)	21 (27.6)		
High	13 (13.1)	5 (6.6)		
Synchronous/metachronous			3.643	0.056
Synchronous	57 (47.5)	49 (61.3)		
Metachronous	63 (52.5)	31 (38.8)		
Extrahepatic metastasis			0.438	0.508
No	79 (65.8)	49 (61.3)		
Yes	41 (34.2)	31 (38.8)		
Previous liver resection			0.104	0.747
No	88 (73.3)	57 (71.3)		
Yes	32 (26.7)	23 (28.8)		
Prior chemotherapy			0.111	0.739
No	16 (13.3)	12 (15.0)		
Yes	104 (86.7)	68 (85.0)		
Primary tumor invasion (missing, *n* = 9)			0.056	0.813
T1–3	72 (63.7)	51 (65.4)		
T4	41 (36.3)	27 (34.6)		
Comorbidities (missing, *n* = 1)			0.709	0.400
No	62 (51.7)	36 (45.6)		
Yes	58 (48.3)	43 (54.4)		
CEA (ng/ml)			0.119	0.730
≤ 30	92 (76.7)	63 (78.8)		
> 30	28 (23.3)	17 (21.3)		
CA199 (0–37 U/mL)			0.104	0.747
Normal	86 (71.7)	59 (73.8)		
Abnormal	34 (28.3)	21 (26.3)		
Primary tumor nodal status			4.409	0.110
Negative	54 (45.0)	25 (31.3)		
1–3	35 (29.2)	33 (41.3)		
> 3	31 (25.8)	22 (27.5)		
Adjuvant chemotherapy post‐ RFA			0.046	0.830
No	24 (20.0)	17 (21.3)		
Yes	96 (80.0)	63 (78.8)		
Major complications			0.136	0.712
No	106 (88.3)	72 (90.0)		
Yes	14 (11.7)	8 (10.0)		

*Note:* The data represent the number of patients, with percentages in parentheses, as well as the results from Pearson's chi‐squared test (*χ* ^2^) for counts data. Measured data are expressed as means ± standard deviations, and t‐test results are presented for measurement data.

Abbreviations: BMI, body mass index; CA19‐9, carbohydrate antigen 19‐9; CEA, carcinoembryonic antigen; cRFA, complete radiofrequency ablation.

### 3.2 Univariate and Multivariate Analysis for Residual Tumor

In our study, based on DCE‐CT/MR findings, 39 cases of CLM (9.5%) were identified as residual tumors, prompting repeat RFA therapy. Among these patients, 246 tumors from 120 individuals were randomly assigned to the development cohort, with a residual tumor incidence of 24 (9.8%). The internal validation cohort included 164 tumors from 80 patients, with a residual tumor incidence of 15 (9.1%). In the development cohort of 246 CLMs, the mean minimal ablative margin was 5.8 ± 2.5 mm, and the mean ∆CT value was 21.8 ± 12.3 HU. Ninety‐one tumors (37.0%) were located near critical organs (e.g., the diaphragm, gastrointestinal tract, and gallbladder); 32 (13.0%) were in perivascular locations; 53 (21.5%) had a diameter ≥ 20 mm; and 129 (52.4%) were subcapsular metastases.

**FIGURE 2:**
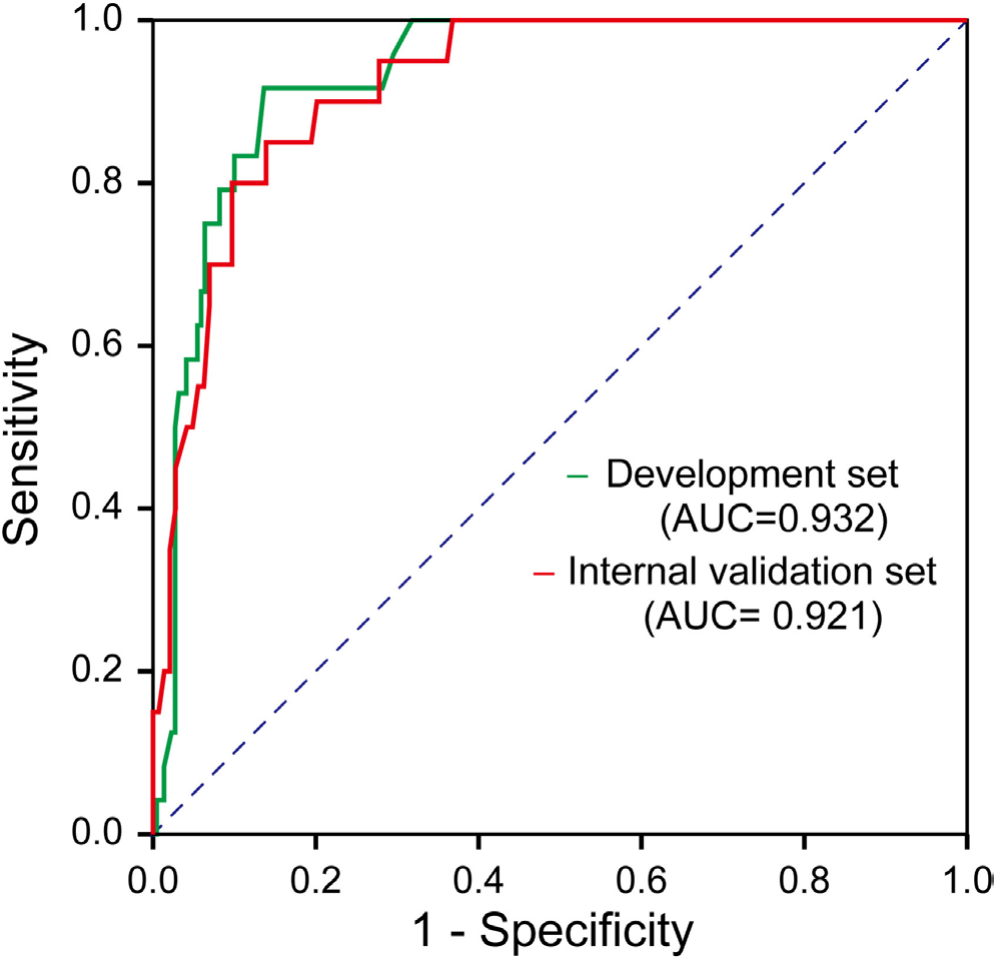
Receiver operating characteristic (ROC) curves for the multivariate logistic regression model in the development (green) and internal validation cohorts (red), with the area under the curve (AUC) corresponding to 0.932 and 0.921, respectively.

Univariate analysis identified the following potential risk factors for inclusion in the multivariate analysis: perivascular tumor location (*p* < 0.001), tumor size ≥ 20 mm (*p* < 0.001), minimal ablative margin (*p* < 0.001), and ∆CT value (*p* = 0.074) (Table 2). MLR analysis (Table 2) showed that perivascular tumor location (odds ratio [OR] = 6.673, 95% confidence interval [CI]: 2.162–20.597, *p* = 0.001), tumor size ≥ 20 mm (OR = 3.925, 95% CI: 1.298–11.869, *p* = 0.015), and minimal ablative margin (OR = 0.599, 95% CI: 0.444–0.807, *p* = 0.001) were independent predictors of residual tumor. The omnibus test confirmed that the model significantly outperformed the null model (*χ* ^2^ = 59.406, *p* < 0.001). Additionally, the Hosmer–Lemeshow test demonstrated good model fit (*χ* ^2^ = 4.991, *p* = 0.759). Figure 2 illustrates the predictive model's performance in both the development and internal validation cohorts, with corresponding AUC values of 0.932 (95% CI: 0.893–0.970, *p* < 0.001) and 0.921 (95% CI: 0.871–0.971, *p* < 0.001), respectively.

**TABLE 2:** Univariable and multivariate analyses of predictors for residual tumor in the development data set.

	Univariable logistic regression			Multivariable logistic regression		
	B	OR (95% CI)	*p*	B	OR (95% CI)	*p*
Far from special locations	−0.386	0.680 (0.270–1.708)	0.411			
Perivascular	2.991	19.902 (7.596–52.146)	< 0.001	1.898	6.673 (2.162, 20.597)	0.001
Subcapsular	0.246	1.278 (0.544–3.001)	0.573			
Size ≥ 20 (mm)	2.245	9.444 (3.819–23.357)	< 0.001	1.367	3.925 (1.298, 11.869)	0.015
∆CT (Hu)	−0.035	0.966 (0.929–1.003)	0.074			
Minimal ablative margin (mm)	−0.677	0.508 (0.389–0.664)	< 0.001	−0.513	0.599 (0.444, 0.807)	0.001

Abbreviations: ∆CT, the difference in CT values between the portal vein and plain scan phase; B, β regression coefficient; CI, confidence interval; OR, odds ratio; special locations, the locations of CLMs that were adjacent to an organ or structure that could affect the ablation procedure, such as the diaphragm, gastrointestinal tract, and gallbladder.

### 3.3 LTPFS

In this study, 64 cases of LTP (26.0%) were observed, with a median LTPFS of 15 months. Univariate and multivariate Cox proportional hazards regression analyses showed that perivascular tumor location (hazard ratio [HR] = 1.911, 95% CI: 1.003–3.644, *p* = 0.049), tumor size ≥ 20 mm (HR = 3.236, 95% CI: 1.750–5.984, *p* < 0.001), and minimal ablative margin (HR = 0.780, 95% CI: 0.688–0.886, *p* < 0.001) were independent predictors of LTPFS. Univariate and multivariate Cox proportional hazards regression analyses indicated that perivascular tumor location (hazard ratio [HR] = 1.911, 95% CI: 1.003–3.644, *p* = 0.049), tumor size ≥ 20 mm (HR = 3.236, 95% CI: 1.750–5.984, *p* < 0.001), and minimal ablative margin (HR = 0.780, 95% CI: 0.688–0.886, *p* < 0.001) independently predicted LTPFS.

Among the 64 CLMs with LTP, 54 (84.4%) underwent further interventional therapy, achieving a median survival of 35 months, while 10 (15.6%) received radiation therapy combined with chemotherapy, with a median survival of 28 months. Kaplan–Meier analysis showed no statistically significant difference (*χ* ^2^ = 0.002, *p* = 0.963). Fourteen patients (21.9%) underwent RFA, with a median survival of 34 months (95% CI: 26.894–41.107). Twelve patients (18.8%) received TACE with a single drug‐loaded microsphere combined with irinotecan, achieving a median survival of 33 months (95% CI: 24.684–41.316). Ten patients (15.6%) received HAIC with the mFOLFOX6 regimen (oxaliplatin: 85 mg/m^2^ for 2 h on Day 1; calcium folinate: 200 mg/m^2^ for 2 h on Day 1; fluorouracil: 400 mg/m^2^ as a bolus on Day 1, followed by 2400 mg/m^2^ over 46 h), achieving a median survival of 31 months (95% CI: 22.718–39.282). Eighteen patients (28.1%) underwent HAIC with the mFOLFOX6 regimen after TACE (irinotecan) and continued treatment in the ward, achieving a median survival of 38 months (95% CI: 32.849–43.151). The log‐rank test showed no statistically significant difference among the various interventional therapy regimens in terms of OS for patients with residual CLMs (*χ* ^2^ = 4.325, *p* = 0.360).

### 3.4 NIHM

**FIGURE 3:**
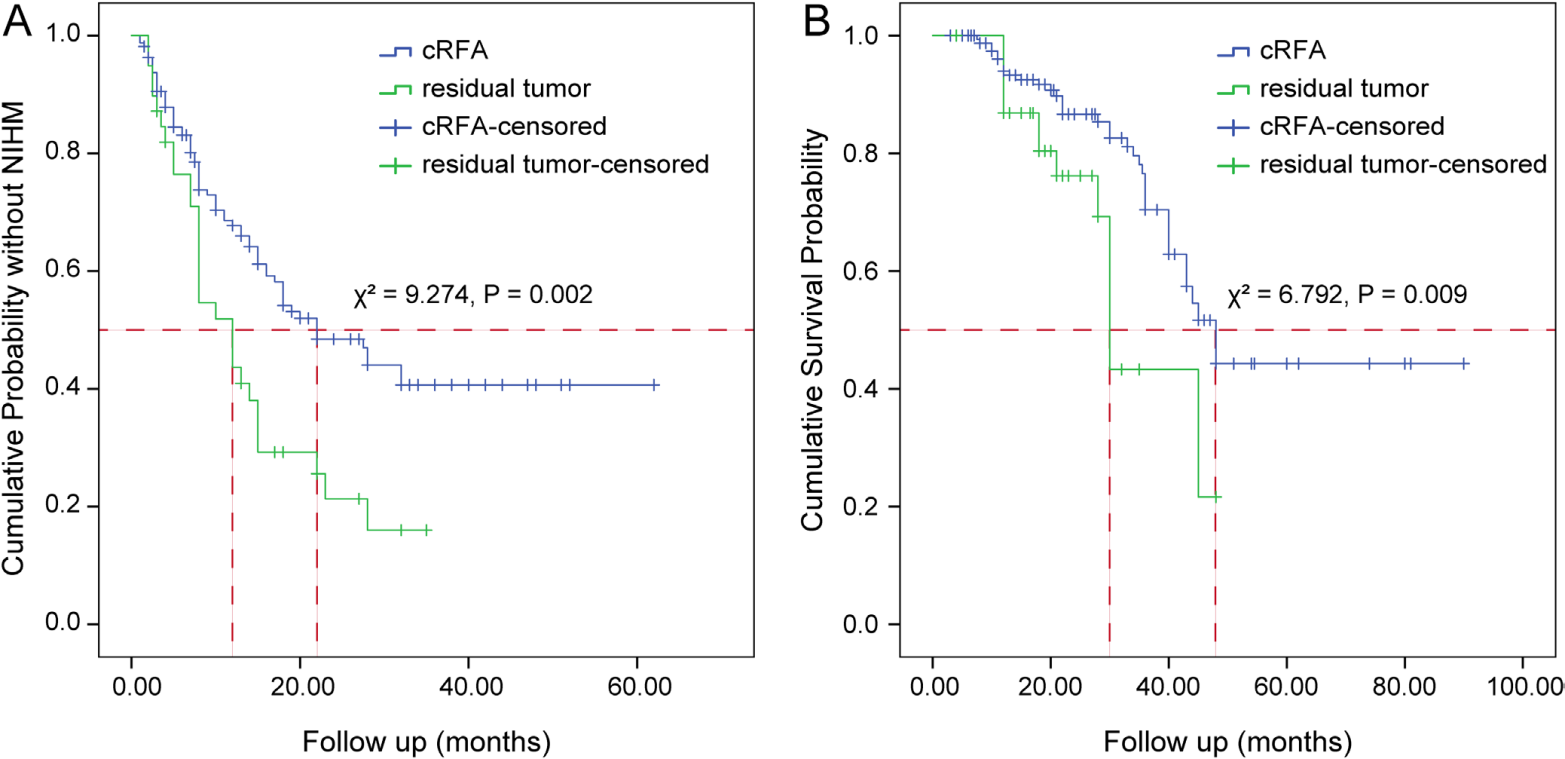
The Kaplan–Meier curves and log‐rank (Mantel–Cox) tests for new intrahepatic metastases (NIHM)‐free survival and overall survival (OS) in the residual tumor group and the complete RFA (cRFA) group. Compared to patients in the residual tumor group, those in the cRFA group had significantly longer NIHM‐free survival (P = 0.002) (A) and OS (P = 0.009) (B).

During the follow‐up period, 98 patients (49.0%) were observed to experience NIHM. The estimated cumulative incidence of NIHM at 1 and 3 years after RFA was 33.0% and 49.0%, respectively. The median NIHM‐free survival time, estimated using Kaplan–Meier analysis, was 18 months (95% CI: 13.795–22.205). In the cRFA group (*n* = 161), NIHM occurred in 69 (42.9%) patients, with a median NIHM‐free period of 22 months (95% CI: 14.731–29.269). In contrast, in the residual tumor group (*n* = 39), NIHM was detected in 29 patients (74.4%), with a median NIHM‐free period of 12 months (95% CI: 9.071–14.929). The difference between the two groups was significant (*χ* ^2^ = 9.274, *p* = 0.002) (Figure 3A).

Appropriate anti‐tumor treatments were administered for NIHM based on each patient's condition, including repeated RFA (*n* = 18), TACE (*n* = 15), HAIC (*n* = 17), TACE + HAIC (*n* = 22), stereotactic body radiotherapy (SBRT) (*n* = 10), and other comprehensive treatments (*n* = 16). Among patients with NIHM who received SBRT, TACE, HAIC, RFA, and TACE + HAIC, the median OS times were 28 months (95% CI: 25.108–30.892), 31 months (95% CI: 22.873–39.127), 36 months (95% CI: 31.765–40.235), 34 months (95% CI: 30.848–37.152), and 43 months (95% CI: 33.902–52.098), respectively. Pairwise Kaplan–Meier analysis and log‐rank tests were performed to compare OS among patients receiving different treatment modalities. The results indicated that OS in the TACE + HAIC group was significantly longer than in the RFA group (*χ* ^2^ = 4.281, *p* = 0.039) (Table 3). However, pairwise comparisons among the other groups showed no statistically significant differences (*p* > 0.05).

**TABLE 3:** Pairwise Kaplan–Meier analysis and log‐rank tests revealed differences in OS among NIHM patients undergoing different treatment modalities.

Groups	SBRT		TACE		HAIC		RFA		TACE+HAIC	
Log‐rank	*χ* ^2^	*p*	*χ* ^2^	*p*	*χ* ^2^	*p*	*χ* ^2^	*p*	*χ* ^2^	*p*
SBRT			0.024	0.877	0.601	0.438	0.221	0.638	0.434	0.510
TACE	0.024	0.877			0.791	0.374	0.063	0.801	2.736	0.098
HAIC	0.601	0.438	0.791	0.374			1.582	0.208	0.107	0.744
RFA	0.221	0.638	0.063	0.801	1.582	0.208			4.281	0.039
TACE+ HAIC	0.434	0.510	2.736	0.098	0.107	0.744	4.281	0.039		

Abbreviations: HAIC, hepatic arterial infusion chemotherapy; NIHM, new intrahepatic metastases; OS, overall survival; RFA, radiofrequency ablation; SBRT, stereotactic body radiotherapy; TACE, transarterial chemoembolization.

### 3.5 OS

The median follow‐up period was 22 months. Among the 200 patients, 50 (25.0%) died, and the cumulative OS rates at 1, 3, and 5 years after RFA were 99.0%, 65.6%, and 40.7%, respectively. The median estimated survival time, calculated using the Kaplan–Meier method, was 45 months (95% CI: 39.354–50.646) for the entire cohort. Log‐rank test results indicated a significant difference in median OS between the cRFA group (48 months, 95% CI: 42.165–53.835) and the residual tumor group (30 months, 95% CI: 27.997–32.003) (*χ* ^2^ = 6.792, *p* = 0.009) (Figure 3B). Based on a competing risk analysis, we adjusted for extrahepatic metastasis (HR = 2.832, 95% CI: 1.050–7.635, *p* = 0.040) and sex (male, HR = 2.251, 95% CI: 1.286–3.941, *p* = 0.010). Subsequently, multivariate Cox proportional hazards regression did not identify any predictive factors for OS.

## 4 Discussion

In this study, we identified three independent factors associated with residual tumor and LTPFS outcomes. Our findings suggest that maintaining an adequate minimal ablative margin helps reduce the likelihood of residual tumor formation. Additionally, we found that a tumor size of ≥ 20 mm and a perivascular tumor location are independent risk factors for residual tumor occurrence after ablation. These results suggest that residual tumor presence may significantly impact both NIHM occurrence and OS.

Adequate ablation margins are crucial for achieving local tumor control and improving long‐term patient survival [6, 8, 18, 19], a conclusion supported by biopsy evidence from previous studies [21]. Several studies have indicated that an ablation margin of ≤ 5 mm is a significant predictor of shorter LTPFS, whereas tumors with margins > 10 mm have shown no observed LTP [10, 11]. Therefore, based on these findings, efforts should be made to ensure that ablation margins exceed 5 mm, with an optimal target of > 10 mm [22]. The critical importance of achieving an adequate minimal margin has been well established, as it has been consistently shown to be one of the most crucial technical endpoints related to the success of ablation [19, 21, 22]. In fact, a minimal margin is a key requirement when ablation is performed with curative intent [19, 21, 22]. Kurilova's study indicated that in patients with a history of HAIC, biliary dilation, and bevacizumab use, a minimal ablative margin > 10 mm is a predictor of biliary complications [23]. The incidence of major biliary complications following RFA was 4% for a minimal ablative margin of 6–10 mm and 21% for margins > 10 mm, with corresponding LTP rates of 24% and 0%, respectively [23]. Therefore, selecting an optimal minimal ablative margin is crucial for balancing local tumor control with the risk of major biliary complications in these patients [23]. However, several previous studies have found that measuring the minimal ablative margin is poorly reproducible and unreliable for predicting treatment success [24]. Recently, Laimer et al. [25] demonstrated that quantifying ablation margins using 3D software based on pre‐ and post‐ablation DCE‐CT images obtained during the intervention is essential for assessing LTP. Additionally, a study by Lin et al. demonstrated that biomechanical deformable image registration and autosegmentation on CT images can effectively identify CLMs at risk for LTP after ablation [26]. A systematic review and meta‐analysis of 21 studies showed that when 3D software confirmed an ablation margin of < 5 mm, the risk of LTP increased 5.1‐fold [22]. This evaluation method offers a high degree of accuracy and enables immediate re‐ablation of inadequately covered areas within a single intervention, potentially enhancing local control rates [27]. Therefore, compared to earlier 2D methods, 3D software provides superior predictive value for assessing the minimal ablation margins at various time points, including pre‐ablation, intra‐procedural, and 4–8 weeks post‐ablation [28, 29]. Kamarinos et al. [29] demonstrated that 3D evaluation with intra‐procedural scans provides a more precise assessment of ablation margins, offering higher sensitivity compared to traditional 2D manual methods. Automated segmentation and measurement using 3D software not only enhance efficiency but also clearly visualize the spatial relationship between the ablation zone and the tumor, facilitating more timely clinical decision‐making [29]. However, the retrospective design and the lack of pre‐contrast‐enhanced scans limit the generalizability of these findings [29]. Additionally, the impact of tissue contraction on ablation volume, particularly after microwave ablation, warrants further investigation [29]. Despite these limitations, the primary advantage of 3D technology is its ability to provide more comprehensive margin information. Future studies incorporating additional imaging techniques and prospective designs could further enhance evaluation accuracy and reliability.

In a recent study [8], perivascular tumor location demonstrated significance in univariate analysis but did not retain significance in the final predictive model after multivariate analysis. Our study underscores that both perivascular tumor location and tumor size are critical factors influencing the risk of residual tumors, highlighting the importance of considering real‐time heat distribution during RFA procedures for tumors of varying sizes. Larger tumors, which are more susceptible to incomplete ablation, were associated with poorer overall survival outcomes. Among the 200 patients in our study, 17 (8.5%) had more than three tumors, and of the 410 CLMs, 29 (7.25%) were larger than 3 cm. In clinical practice, RFA is performed after assessing liver and kidney function, coagulation parameters, and imaging studies, with informed consent obtained from the patient and their family. Advances in imaging modalities and ablation techniques have broadened the eligibility criteria for RFA. Most studies suggest that the optimal tumor size for ablation is < 3 cm, a conclusion supported by Shady et al., who identified tumor size > 3 cm as a key criterion for high‐risk patients using a modified risk score [6]. While some retrospective studies have primarily focused on tumors < 3 cm, with only a limited number > 5 cm, they consistently highlight the association between tumors > 3 cm and an increased risk of LTP after ablation [6, 30].

Additionally, Dijkstra et al. found no significant difference in OS or complication rates between patients with CLMs of 3–5 cm and those with CLMs of 0–3 cm treated with thermal ablation. However, the 0–3 cm group exhibited better local tumor control and longer local tumor progression‐free survival, consistent with the findings of this study [31]. Furthermore, an international panel of ablation experts reached a consensus that well‐positioned tumors ≤ 5 cm could be effectively treated, depending on their anatomical location and treatment protocol [32]. Ablation was routinely recommended for patients with five or fewer tumors, supporting the inclusion criteria of this study [32]. In clinical practice, however, when tumors exceed 3 cm or when more than three tumors are present, a more comprehensive and cautious decision‐making process is necessary [32]. The anatomical location of the tumor, its proximity to critical structures, and the complexity of the procedure are key factors in determining the feasibility and safety of ablation. Larger tumors, particularly those near the liver capsule or major vessels, are associated with a higher risk of complications and incomplete ablation. Therefore, a multidisciplinary team approach, incorporating advanced imaging techniques and a thorough assessment of the patient's overall condition, is essential for determining the optimal treatment strategy.

We achieved favorable outcomes by performing repeated interventional treatments for certain recurrent and newly developed tumors, particularly through the use of TACE and HAIC in patients with NIHM. Although previous meta‐analyses have suggested the superiority of RFA combined with systemic chemotherapy [33], limited research exists on the combination of RFA with localized chemotherapy (TACE/HAIC), highlighting the need for further investigation in this area. Notably, while extrahepatic metastasis can influence OS, this variable did not significantly affect outcomes in the residual tumor versus cRFA groups (*p* > 0.05) in our study. A prospective randomized phase II trial demonstrated that patients with unresectable CLMs who received systemic therapy in combination with RFA or resection had significantly longer OS than those who underwent systemic therapy alone (45.6 months vs. 40.5 months, *p* = 0.01) [34]. These findings suggest that patients with advanced CLMs may benefit from aggressive local treatment strategies. Moreover, to meet statistical requirements, at least 8–10 samples were allocated per variable in the regression model. Postoperative imaging assessments revealed a small amount of pleural effusion in three patients; however, none exhibited severe symptoms, such as respiratory distress, and all recovered without specific treatment. Two cases of pleural effusion were attributed to the proximity of CLMs to the diaphragm, while the third resulted from the puncture trajectory during the RFA procedure. Similarly, a study by Dimopoulos et al. [35] demonstrated that adverse events following percutaneous MWA in patients with CLMs included pneumothorax, which was significantly associated with proximity to the diaphragm (*p* < 0.001) and the transpleural approach (*p* < 0.001). Although pneumothorax prolonged the hospital stay, it did not result in any long‐term complications. Including only patients with resected primary tumors enhances study consistency and reduces confounding factors related to CLM progression and prognosis. Patients with unresected primary tumors may experience poorer local control of liver metastases, potentially affecting efficacy assessments. Thus, focusing on resected cases enables a more accurate assessment of ablation outcomes and long‐term survival, while also aligning with common clinical practice where primary tumors are typically resected before RFA for CLMs.

Several limitations of this study should be acknowledged. First, the cohort used to develop the prediction model was relatively small, leading to a model with fewer variables, which may constrain its predictive accuracy for residual tumors. Second, the lack of extensive external validation datasets raises concerns about the generalizability of our model to other regional or international populations. Finally, factors such as the neutrophil‐to‐lymphocyte ratio [36] and genetic mutations (e.g., KRAS, RAS, NRAS, or dMMR/MSI‐H) [37, 38, 39], which may influence NIHM and OS, were not comprehensively analyzed or adequately adjusted due to incomplete data.

In conclusion, we precisely measured the minimal ablative margin in RFA procedures and successfully developed and validated a residual tumor prediction model with robust performance. An adequate minimal ablative margin was identified as an independent protective factor against residual tumors and LTPFS, while tumor size ≥ 20 mm and perivascular tumor location were identified as independent risk factors. Residual tumors may contribute to the occurrence of NIHM and poorer OS outcomes. This predictive model could aid clinical decision‐making in tailoring treatment strategies for patients with CLMs based on tumor characteristics.

## Author Contributions

All authors made significant contributions to the conception and design of the study. H.L., X.X., and Y.Z. contributed equally to this work. H.L., Y.Z., X.X., X.X., X.D., F.B., and H.F. were responsible for material preparation, data collection, and analysis. The initial draft of the manuscript was written by H.L. and X.X., H.F. and S.X. were responsible for data curation, formal analysis, investigation, conceptualization, writing review, and editing. All authors contributed to manuscript revision, read, and approved the submitted version.

## Ethics Statement

The study was approved by the Ethics Review Committee of Quzhou Affiliated Hospital of Wenzhou Medical University (Approval No. 2023‐269). Consent was obtained from all participants and/or their legal guardians, and all methods were carried out in accordance with the ethical standards of the responsible committee on human experimentation and with the Declaration of Helsinki.

## Consent

The authors have nothing to report.

## Conflicts of Interest

The authors declare no conflicts of interest.

## Supporting information


Table S1.

## Data Availability

The datasets used and/or analyzed during the current study are available from the corresponding author on reasonable request.
