# Supplementary material for: Prognostic Comparison of Complete vs. Incomplete Radiofrequency Ablation for Colorectal Liver Metastases: A Multicenter Prospective Study
Source: Cancer Med. 2025 Apr 15;14(8):e70735. doi: 10.1002/cam4.70735 (PMC11997704; doi:10.1002/cam4.70735)
Supplement: Supplementary file 1 — Table S1. [file CAM4-14-e70735-s001.docx]

Supplementary Table Scan and the reconstruction parameters of computed tomography.

| The scan parameters |  |
| --- | --- |
| X-ray tube voltage | 120 kV |
| X-ray tube current | 80 mA |
| rotation time | 0.5 s |
| layer spacing | 5 mm |
| layer thickness | 5 mm |
| Pitch | 0.984 |
| Matrix | 512×512 |
| The reconstruction parameters |  |
| layer thickness | 1.25 mm |
| layer spacing | 1.25 mm |
| reconstruction algorithm | Standard |
| reconstruction window | Abdomen |
